# Adaptation to Aridity in the Malaria Mosquito *Anopheles gambiae*: Chromosomal Inversion Polymorphism and Body Size Influence Resistance to Desiccation

**DOI:** 10.1371/journal.pone.0034841

**Published:** 2012-04-13

**Authors:** Caroline Fouet, Emilie Gray, Nora J. Besansky, Carlo Costantini

**Affiliations:** 1 Institut de Recherche pour le Développement (IRD), UMR MIVEGEC (UM1, UM2, CNRS 5290, IRD 224), Montpellier, France; 2 Organisation pour la Coordination de la lutte contre les Endemies en Afrique Centrale (OCEAC), Yaoundé, Cameroon; 3 Eck Institute for Global Health, Department of Biological Sciences, University of Notre Dame, Notre Dame, Indiana, United States of America; Instituto de Higiene e Medicina Tropical, Portugal

## Abstract

Chromosomal inversions are thought to confer a selective advantage in alternative habitats by protecting co-adapted alleles from recombination. The frequencies of two inversions (2L*a* and 2R*b*) of the afro-tropical malaria mosquito *Anopheles gambiae* change gradually along geographical clines, increasing in frequency with degree of aridity. Such clines can result from gene flow and local selection acting upon alternative karyotypes along the cline, suggesting that these inversions may be associated with tolerance to xeric conditions. Since water loss represents a major challenge in xeric habitats, it can be supposed that genes inside these inversions are involved in water homeostasis. To test this hypothesis, we compared the desiccation resistance of alternative karyotypes from a colonised 2R*b*/2L*a* polymorphic population of *A. gambiae* from Cameroon. The strain included only the molecular form S, one of the genetic units marking incipient speciation in this taxon. Day-old mosquitoes of both sexes were assayed individually for time to death in a dry environment and the karyotype of each was determined *post-mortem* using molecular diagnostic assays for each inversion. In agreement with expectations based on their eco-geographical distribution, we found that 2L*a* homokaryotypes survived significantly longer (1.3 hours) than the other karyotypes. However, there was weak support for the effect of 2R*b* on desiccation resistance. Larger mosquitoes survived longer than smaller ones. Median survival of females was greater than males, but the effect of sex on desiccation resistance was weakly supported, indicating that differential survival was correlated to differences between sexes in average size. We found weak evidence for a heterotic effect of 2L*a* karyotype on size in females. These results support the notion that genes located inside the 2L*a* inversion are involved in water balance, contributing towards local adaptation of *A. gambiae* to xeric habitats, beyond the adaptive value conferred by a larger body size.

## Introduction

Population characteristics including morphology, phenology, and stress resistance commonly feature gradual changes in time or space associated with gradients in environmental variables such as temperature, rainfall, altitude, or insulation [1], [2]. Such clines have been extensively studied in plants and animals, and are known to occur at different geographical or ecological scales. While the reasons for the existence of such clines are often obvious, the underlying mechanisms that generate and maintain them are still poorly understood. One possible mechanism is through paracentric chromosomal inversions, which capture allelic combinations favourable to a particular environment [3]–[6]. Many studies have found geographical clines in chromosomal inversion frequencies within broadly distributed species [7]. Investigations of natural populations of *Drosophila* in particular have demonstrated the existence of latitudinal clines in inversion frequency associated with traits as body size, developmental time, and heat or cold tolerance [8]–[11].

Paracentric chromosomal inversions are thought to emerge and spread in natural populations because they protect favourable allelic combinations from recombination. Clines of polymorphic inversions can result from the combined action of gene flow and multilocus local selection of alleles protected from recombination by the inversion [12]. However, other processes can also produce clinal patterns. Secondary contact of allopatric populations fixed for alternative chromosomal arrangements, or genotype-dependent dispersal offer suitable alternative explanations [13], [14]. It is difficult, therefore, to disentangle the independent effects of population history and selection without first assessing the fitness advantage of alternative inversions in different environments.

Chromosomal inversions are widespread among members of the *Anopheles gambiae sensu lato* (*s.l*.) complex of sibling species, some of which are the most important vectors of human malaria in sub-Saharan Africa [15]. In this complex, paracentric inversions are found both as fixed genetic markers differentiating the species and as floating polymorphisms within species [15]. It has been hypothesized that inversion polymorphisms may be responsible for much of the adaptive ecological potential in this species complex [15], [16]. The chromosomal polymorphisms with the largest geographical distribution are those involving inversions on the left and right arm of chromosome 2 (the 2L*a* and 2R*b* arrangements, respectively); these have been extensively studied in *A. gambiae sensu stricto* (*s.s*.), and found to correlate with factors such as aridity [17], [18], and insecticide resistance [19].

In a seminal paper published more than 30 years ago, Coluzzi and colleagues reported the existence of a latitudinal cline in the frequency of inversions 2R*b* and 2L*a* in Nigeria, from coastal mangroves and the humid rainforest of the south, up to the pre-desertic Sahelian steppe in the north [17]. The inverted arrangements were fixed or almost so in the most xeric habitats, whereas the standard (i.e. non-inverted) arrangements prevailed in mesic environments. The two inversions changed clinally in frequency along this geographical/aridity gradient. More thorough and extensive surveys across Africa have confirmed those observations (e.g. [20]) and the relationship between the frequency of the 2L*a* inversion and degree of aridity has been formally quantified [18], [21]. Furthermore, the 2L*a*-inverted arrangement is fixed in another member of the *A. gambiae s.l*. complex, i.e. *A. arabiensis*, which is adapted to live in xeric habitats [22], [23]. From these studies, it was suggested that carriers of the 2L*a* arrangement bear some selective advantage in xeric habitats compared to standard karyotypes. The functional mechanisms underlying such adaptive value have been difficult to assess until recently due to the technical limitations inherent in karyotyping *Anopheles* mosquitoes by traditional cytogenetic techniques. The recent development of DNA-based assays to score the chromosomal status of inversions in individual mosquitoes regardless of their developmental stage or sex [24]–[26] has opened new approaches in the study of the ecological genetics of this medically important group of insects.

In Africa, insects are faced with several important challenges in xeric habitats due to the occurrence of higher temperatures, greater solar radiation, and drier conditions than those encountered in mesic environments. Specific physiological adaptations or behavioural strategies, therefore, should evolve to cope with the thermal and dehydration stresses imposed by xeric habitats. Given the strong correlation between 2L*a* or 2R*b* frequency and degree of aridity, it can be supposed that genes inside or near the breakpoints of these inversions may be involved with specific homeostatic responses to counteract the detrimental effects of thermal and/or dehydration stress. Indeed, research on *A. gambiae* larvae has revealed enhanced resistance to thermal stress in carriers of the 2L*a*-inverted arrangement following heat hardening (exposure to transient sub-lethal temperature) [27]. The heat hardening transcriptional response involves the up-regulation of *hsp* gene families responsible for molecular chaperoning, proteolysis function, and energy metabolism [28]; further examination of these populations found the proportion of up-regulated *hsp* genes to be much higher in 2L*a*-inverted individuals compared with 2L*a*-standard individuals [28]. Similarly, adult females carrying the 2L*a* inverted arrangement were more resistant to desiccation, owing to lower rates of water loss at emergence, and higher body water content at 4 days post-emergence [29].

In this work, we provide additional evidence for the association between the 2L*a* chromosomal inversion and resistance to desiccation in *A. gambiae* from a recently colonized polymorphic population collected in central Cameroon. This extends the work of Gray *et al.* [29] in several important ways. Aside from chromosomal inversion polymorphisms, *A. gambiae s.s*. has also been subdivided based on fixed differences in rDNA [30]: two molecular forms–named M and S–are strongly differentiated genetically [31], [32], and ecologically [20], [33], [34]. This suggests that they are evolving along independent trajectories, yet the evolution of reproductive isolation between them is not complete [35]. Previous studies on the effects of the 2L*a* inversion on stress resistance have used laboratory strains of the M form [27]–[29]. However, because of their discontinuous geographical distribution [20], [36], [37], natural populations of the M form are almost fixed for either the inverted or the standard 2L*a* arrangement. As a result, studies on populations of the M form may fail to disentangle the effects of 2L*a* status and other geographically-dependent genetic differences. Conversely, the 2L*a* polymorphism follows a cline in natural populations of the S form across its continuous distribution from the rainforest to the arid savannas of Western and Central Africa [20], [33], [38]. Thus, studying a recently colonised S population polymorphic for 2L*a* and 2R*b* enables us to observe not only the differential responses of heterokaryotypes of each inversion but also the effect of different genetic backgrounds on each inversion.

To achieve this aim, we have assessed the degree of resistance to desiccation of day-old *A. gambiae* individuals by measuring their survival in dry air. All individuals tested originated from one large polymorphic colony, and their karyotype status was identified for the 2L*a* and 2R*b* inversions. Since body size represents an important correlate of desiccation resistance [39], we used a proxy for body size to assess whether size and karyotype interact to increase survival under dehydration stress. Last but not least, as males and females have been shown to differ both in the degree of desiccation resistance [40], and body size [41], we disentangled the separate effects of size and sex upon desiccation resistance to understand whether epistatic effects might be responsible of the observed differences between sexes.

## Results

### Inversion 2Rb and Desiccation Resistance

**Figure 1:**
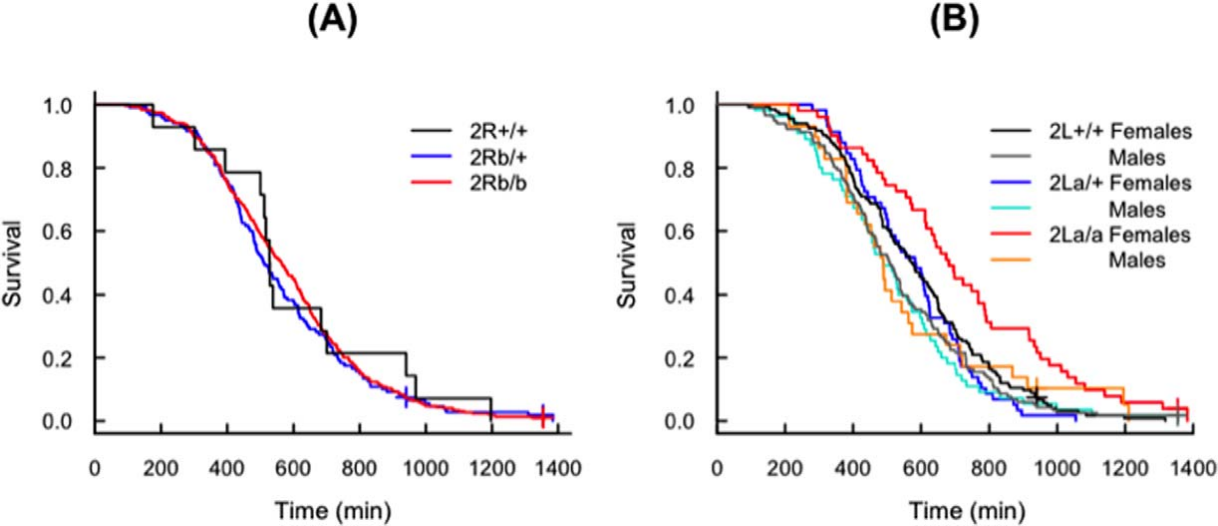
Survival of *Anopheles gambiae* mosquitoes submitted to desiccation stress. Kaplan-Meier survivorship curves stratified according to (A) 2R*b* inversion karyotype; (B) sex and 2L*a* inversion karyotype.

The survival of carriers of different 2R*b* arrangements under dehydration stress was similar (Fig. 1A), although 2R*b*/*b* individuals had the highest median survival (*c.* 0.5 hrs greater than the other two 2R*b* arrangements; Table 1). This result must be gauged with caution because of the small sample size of 2R*b*-standard karyotypes, and the non-negligible error rate of the 2R*b* molecular diagnostic test [26]. Therefore, our results indicate there may be a modest effect of the 2R*b* inversion on desiccation resistance. Because this effect is minor, we do not take into account the 2R*b* karyotype status in the remainder of the text.

**Table 1:** Summary statistics of *Anopheles gambiae* survival under experimental dehydration stress.

Variable		Sample size	Censored	Median survival (min)	95% CI
Karyotype	2L+/+	250	2	537	506–575
	2La/+	113	1	529	490–600
	2La/a	80	2	612	542–697
	2R+/+	14	0	528	512–970
	2Rb/+	120	2	513	480–570
	2Rb/b	309	3	564	524–605
Sex	Females	243	2	603	565–630
	Males	200	3	500	464–533
Wing length	Very Small	117	4	498	403–561
	Small	117	0	494	462–540
	Large	100	0	526	487–609
	Very Large	109	1	635	612–691

Median survival times and their 95% confidence limits were estimated for several stratifying variables. Sample size is the number of tested mosquitoes falling under each category. Censored observations represent the number of individuals that were still alive at the end of the experimental period. Wing length was subdivided in four classes corresponding to intervals of length defined by quartiles: Very Small [0%, 25%], Small [25%, 50%], Large [50%, 75%], and Very Large [75%, 100%].

### Inversion 2La and Desiccation Resistance

**Figure 2:**
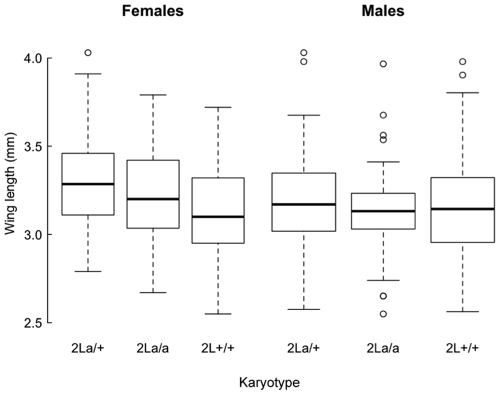
Wing length of *Anopheles gambiae* tested for desiccation resistance, stratified by sex and 2L*a* karyotype. Thick horizontal lines in the box-whisker plots represent medians, boxes define the interquartile range, and vertical dotted lines delimit the range of wing lengths. Points represent outliers defined as values exceeding the mean ±2×SD.

The relationship between survival and 2L*a* karyotype stratified by sex is shown in Fig. 1B. On average, females survived almost 2 hrs longer than males, and 2L*a* inverted karyotypes survived *c.* 1.3 hrs longer than both standard and heterokaryotypes (Table 1). Mosquito size was positively correlated with survival (Table 1 and Fig. S1A). Additionally, we found correlations between covariables: heterokaryotypic and inverted 2L*a* females were on average larger than standard females, and larger than males regardless of karyotype (Fig. 2). Because of these correlations and of differences in survival across replicates (Fig. S1B), we modelled the survival of tested mosquitoes by Cox proportional hazards regression stratifying for these factors, with the objective to assess the impact of each factor on desiccation resistance adjusted for the effect of other correlated variables. Before doing so, we verified whether wing length was significantly associated with sex and karyotype, as shown in Fig. 2.

### Inversion 2La and Mosquito Size

Several linear mixed models having the factors SEX and KARYOTYPE as fixed effects and REPLICATION as a random effect were evaluated by a set of statistical evaluators (Table S1). The minimal adequate model, as inferred by the lowest Akaike Information Criterion (AIC), was the one having only the factor SEX included as explanatory variable (Model 3 in Table S1). Thus, evidence for a significant association between carriers of the 2L*a* inversion and wing length (only in females) was not strong enough in our data set (discussed in more detail in Text S1), but our results warrant further investigations about this relationship. On the other hand, our data support the inference that males had significantly shorter wings than females, regardless of karyotype.

### Disentangling the Impact of Covariables upon Survival

The Cox proportional hazards model having the lowest AIC was that including KARYOTYPE and SIZE as main effects (Model 6 in Table S2, discussed in more detail in Text S2). The parameter estimates of this model (Table 2) indicate that under dehydration stress the instantaneous risk of death of 2L*a*-inverted karyotypes at any point in time relative to that of heterokaryotypes or 2L*a*-standard karyotypes (i.e. the hazard ratio) was 0.686 (95% confidence interval: 0.501–0.939; *P*<0.02). A hazard ratio significantly lower than unity implies that 2L*a* karyotypes died more slowly than the other karyotypes (as can be seen from the survivorship curves in Fig. 1B), i.e. they showed greater tolerance to desiccation. The hazard ratio of heterokaryotypes and 2L*a*-standard karyotypes was not significantly different from unity, denoting that their degree of desiccation resistance was the same (Table 2). These estimates are adjusted to take into account the effect of size upon survival. Similarly, the instantaneous risk of death of larger individuals was 0.978 (95% confidence interval: 0.959–0.987; *P*<0.001) that of smaller individuals, independent of karyotype, for every unit increase of the derived variable SIZE, obtained as a cubic transformation of wing length. To compare the relative contribution of karyotype status and mosquito size to survival, we defined the region of wing length values for which the effect of karyotype was greater than that of size. This is presented in Fig. S2 and discussed in more detail in Text S3.

**Table 2:** Summary statistics of Cox proportional hazards survival regression analysis.

Parameter	β	SE	z	*P*	Ψ	95% CL
KARYOTYPE:Inverted	–0.37715	0.16027	–2.353	0.0186	0.686	0.501–0.939
KARYOTYPE:Standard	0.00003	0.12529	0.0003	0.9998	1	0.782–1.278
SIZE	–0.02738	0.00718	–3.812	0.0001	0.973	0.959–0.987

Parameter estimates (β) and standard errors of the minimal adequate proportional hazards model (Model No. 6 in Table S2) fitted to the desiccation stress survival data. Values of ψ represent adjusted hazard ratios with respect to 2L*a* heterozygotes (for KARYOTYPE), or unit increases of SIZE (wing length cubed).

## Discussion

Geographical clines of chromosomal inversions offer suitable models to understand the genetic basis of adaptation and the forces shaping and maintaining chromosomal polymorphisms in natural field populations. Here we have examined whether disruptive selection to arid *versus* mesic conditions might explain the clinal frequency of the 2L*a* and 2R*b* chromosomal inversions in the major afro-tropical malaria vector *A. gambiae*. To this aim, we have tested whether there is a significant association between karyotype and time to death under desiccation stress to assess resistance to dehydration, a trait that is likely to be significant for the survival of populations living in the more xeric portions of this species’ extensive geographical range.

### Influence of Body Size on Desiccation Resistance

Organisms living in arid environments are faced with the challenges of water acquisition and conservation. Survival in such conditions can be enhanced by at least 3 physiological mechanisms: (i) accumulation of greater amounts of water, as either free or metabolic body water, e.g. in the form of lipid storage; (ii) decrease in the rate at which water is lost; or (iii) increase in the physiological tolerance to low water content [42], [43]. Insects can use strategies impacting any combination of these mechanisms to achieve water homeostasis.

Water is lost from the insect’s body by excretion, by diffusion through the cuticle, or during respiratory gas exchange through the spiracles [39]. To reduce water loss, therefore, insects can adapt by reducing excretion of fluids, by modifying the quantity and composition of cuticular lipids to decrease cuticular permeability [44], or–perhaps–by optimising the opening of the spiracles [42], but see [43], [45]. Water loss through the body surface decreases as the surface area-to-volume ratio decreases, hence larger insects are better adapted to resist water loss [42], [46]. Analogously, a larger body also allows storage of larger reserves of free body water (in tissues and the haemolymph), as well as metabolic water produced from the catabolism of glycogen and especially lipids [47]. Thus, water conservation requirements may impose a selection pressure for larger body size in xeric climates [39]. Other components of mosquito fitness such as fecundity (ovariole number) and pre-gravid rates [48] are also affected by body size, with larger individuals at an advantage compared to smaller ones.

In agreement with expectations based on the above considerations, in our study we observed that under desiccation stress larger *A. gambiae s.s*. survived longer than smaller ones. The advantages conferred by a larger size could contribute to explain geographical inter-specific variation in insect size [49]. Among the *A. gambiae s.l*. siblings, field-collected samples of *A. arabiensis*, the species of the complex living in xeric and cooler climates [22], [23] and having greater resistance to desiccation [50], consistently show a larger size compared to *A. gambiae* [51]. Our results suggest that, in the absence of other trade-offs, larger individuals would be favoured in more xeric climates, leading to intra-specific variability in body size among *A. gambiae* populations.

### Influence of Genetic Background on Desiccation Resistance

We have demonstrated that teneral *A. gambiae* 2L*a*-inverted homokaryotypes survived significantly longer (*c.* 1.3 hrs) in dry air than heterokaryotypic or homokaryotypic-standard individuals. Likewise, Gray *et al*. [29] found that, under similar experimental conditions, the median survival of teneral female 2L*a*-inverted *A. gambiae* was *c*. 3.1 hrs longer than that of homokaryotypic standard individuals. Decreased water loss rates explained the observed differences in survival of teneral M-form strains tested by Gray *et al*. [29]. Although it is not possible to infer from our results the underlying physiological mechanisms involved in increased desiccation resistance in our S-form population, it seems likely that reduction in rates of water loss might account for the differences among the 2L*a* karyotypes tested. It is worth noting, however, that the degree and nature of desiccation resistance can change with age [29], [50]. In the aforementioned study, the largest difference between 2L*a* karyotypes in the degree of desiccation resistance was observed in teneral mosquitoes (≤1 day old). This is an ontogenetic phase when the cuticle is still hardening [52] and the cuticular hydrocarbon profile is not fully mature [53]. As *A. gambiae* got older, changes in mass-specific water loss rates and initial water content made differences between alternative 2L*a* karyotypes disappear [29]. Selective effects on 2L*a* concerning this trait are therefore likely to operate early on during *A. gambiae* adult life, which justified our choice to work with newly emerged mosquitoes. As the majority of female *A. gambiae* mate only once during the first 2–4 days of adult life [54], early selection for desiccation resistance mediated by the 2L*a* karyotype is also indirectly related to the mating success component of fitness.

Although our study provides only preliminary evidence, it suggests that the effect of the 2R*b* inversion on desiccation resistance is minor or negligible, compared to 2L*a*. Therefore, the latitudinal cline in 2R*b* inversion frequency observed in Nigeria [17] and Cameroon [20] is probably attributable to other factors. Other life history traits associated with or correlated to fitness components in xeric habitats, e.g. thermal tolerance [27], await testing. Moreover, our experiments did not consider hardening responses: prior acclimation to a dry environment can increase desiccation resistance in *A. gambiae* due to a reduction in water loss rates, regardless of 2L*a* karyotype status; physiological changes in lipid and glycogen metabolism resulting from hardening, however, are affected by 2L*a* karyotype [29]. It is possible, therefore, that the 2R*b* inversion may also result in alternative hardening responses to dehydration stress.

Lee *et al*. [40] found that field populations of the M molecular form in Mali survived longer than S under dehydration stress, but they did not test for the separate effects of body size, age, and karyotype. In agreement with this study, comparison of the median survival of our S population with the M population of Gray *et al*. [29], confirms that teneral 2L*a*-inverted M females survived longer than their S counterparts (14.7 *vs*. 11.4 hrs, respectively), although these estimates are not adjusted for possible differences in body size between the two experimental populations. Remarkably, differences in survival persisted also in the case of 2L*a*-standard homokaryotypes (11.6 *vs*. 9.6 hrs, respectively). The M form, therefore, might carry genetic factors other than the 2L*a* (and, perhaps, the 2R*b*) inversion(s) enabling it to resist desiccation more than S. Such a difference conforms with the known geographic distribution of these forms, given that the savanna populations of the M form are proportionally more abundant in the more xeric regions of West Africa, whereas S prevails in more mesic conditions encountered in the humid Guinean savanna [33].

### Influence of Chromosomal Polymorphism on Body Size

Several studies with *Drosophila* have shown that chromosomal inversion polymorphism can affect size-related traits [55], [56], and body shape [57], [58]. In *D. buzzatii*, second chromosome inversions affect thorax length [55], [56], [59], longevity [56], [60], developmental time, and pre-adult viability [61], [62]. Kennington *et al.* [63] found localised genomic regions controlling variation in wing size within the cosmopolitan inversion In(3R)Payne in *D. melanogaster.* In our data set, evidence for a heterotic association between 2L*a* karyotype and wing length was weak, and–if effectively present–it concerned only females. We cannot exclude, however, that the 2L*a* inversion might affect desiccation resistance through indirect effects upon body size. For example, genes within the inversion may influence the rate of growth, the allocation of metabolic resources, or larval developmental time [64]. If so, the effect of 2L*a* karyotype on size may contribute to the maintenance of its polymorphism. The observation that covariation concerned only females suggests that epistatic interactions of genes inside (or close to the breakpoints of) the 2L*a* inversion with sex-specific genes might also exist.

### Influence of Sex on Desiccation Resistance

In agreement with the findings of Lee *et al*. [40], we found that *A. gambiae* females survived longer than males under desiccation stress. Our data suggest that greater desiccation resistance in females may be mostly due to their average larger size compared to males. Sexual size dimorphism is under several selection pressures and is presumably maintained by selection for protandry [65], [66], and mating-recognition systems [67].

### Conclusions and Perspectives

The present study demonstrates a significant association between the 2L*a* chromosomal inversion and resistance to desiccation in the major malaria vector *A. gambiae*. These results support a role of this inversion in water homeostasis, which can explain the clinal pattern of 2L*a* across the range of eco-climatic conditions encountered in Western and Central Africa. Our study also demonstrates the role of body size in increasing survival of this mosquito in dry environments.

This study has investigated the response of different karyotypes under laboratory settings that do not reflect the environmental conditions experienced by *A. gambiae* in nature. The aim was to verify whether inherent differences between karyotypes exist in a physiological response for stress tolerance associated to water homeostasis. Other mechanisms, including behavioural strategies optimizing the intake and loss of water, may contribute to increase the survival of *A. gambiae* in xeric habitats; these should be addressed by further investigations.

The public health significance of the adaptive mechanisms associated with the 2L*a* inversion stems from the enhanced ability of *A. gambiae* to exploit dry habitats and seasons, thereby extending in space and time the transmission of malaria in Africa [68]. The 2L*a* inversion has also been associated with differential adult resting and biting behaviours [17], bearing consequences on the non-uniform exposure of *A. gambiae* to indoor residual insecticides [69], and on *Plasmodium* infection rates [70]. The understanding of the genetic basis of behavioural and ecological traits associated to chromosomal inversions should therefore allow to better predict how *A. gambiae* populations will respond to epidemiologically-relevant environmental changes acting at global scales, as well as to anti-vector measures, and will hopefully help in the design of new strategies for intervention.

## Materials and Methods

### Desiccation Resistance Assays

The degree of resistance to desiccation was assessed by measuring the survival of individual mosquitoes placed in a vial containing a desiccating agent as in [29], [50]. Survival was determined with a video recording system allowing to score *post hoc* the time to death of each mosquito. The video set-up consisted of CCTV cameras connected to a computer equipped with a frame grabber and video recording software (EZ Watch Pro® v. 4.0). The behaviour of up to 50 individual mosquitoes per experimental replicate was video recorded at a resolution of 704×480 pixels and 24 Hz. Time to death was measured with a precision of ±1 min. The room hosting the experimental set-up had a temperature of 26±1°C, with lights continuously switched on during the experiments.

Mosquitoes of both sexes were let emerge in a cage where they had access to a 10% sucrose solution and tested the following day when they were ≤ 24 hours old. Individual mosquitoes were placed in the lower half of 2×10 cm glass vials sealed with Parafilm® and containing a foam rubber stopper and a desiccant (silica gel) in the upper half of the vial. Relative humidity in the bottom half of the vial was 5%, as measured with a Testo™ 435 Multimeter. The experiment started immediately after the desiccant was introduced in the vials, and ended after 24 hrs. Although sexes were not separated at emergence, this protocol should insure that female mosquitoes were still virgin at the time they were tested. Individual mosquitoes were scored as dead when they were knocked-down or otherwise immobile until the end of the experiment. Death was confirmed by visual inspection when mosquitoes were retrieved from the vials for further processing (cf. below). Mosquitoes that at the end of the experiment were still alive were killed and processed as dead ones, but they were considered as censored observations for the purpose of statistical analysis. Twelve experimental replicates were carried out, for a total of 550 mosquitoes tested and successfully karyotyped, overall.

### Mosquitoes

Experiments were carried out with an *A. gambiae s.s.* molecular form S laboratory strain originating from Ndokayo (5°30.723′N 14°07.497′E), a village located in the forest/savanna mosaic transition region of eastern Cameroon. In this area, natural populations of *A. gambiae* are polymorphic for the 2L*a* and 2R*b* chromosomal inversions, with frequencies averaging about 50% for the 2L*a* and 60% for the 2R*b* inversion (our own unpublished data). Our objective was to test the colony as soon as feasible following colonisation in the laboratory to minimize the effect of laboratory acclimation upon water loss rates [71], yet remove parental and grandparental environmental effects possibly confounding the genetic differences between karyotypes. Due to difficulties with mating and female insemination during the early generations of the lab colony–a common and disagreeable problem with *A. gambiae*–sufficient sample sizes were achieved and experiments were performed using F9 to F12 generations.

The colony was kept at ambient conditions in the insectarium of the Malaria Research Laboratory of OCEAC in Yaounde, the capital of Cameroon. During the course of the experiments, the colony remained polymorphic at both inversions with frequencies ranging 11–32% and 39–93% for the 2L*a* and 2R*b*, respectively. Individuals carrying all possible karyotype combinations could be tested for desiccation resistance; however, due to the low frequency of the 2R+*^b^*/+*^b^* arrangement and the fact that the karyotype of individual mosquitoes was not known beforehand, several replicates lacked one or more of the nine karyotype combinations. Thus, the effect of each inversion system was evaluated separately.

Different cohorts of emerging mosquitoes represented separate test replicates. Because of heterogeneities in larval growth and development, we did not make specific efforts to control for the sex ratio and size of the tested mosquitoes, which could account at least partly for the variability in results across replicates (cf. Figures S1B).

### Species Identification and Molecular Karyotyping

To confirm the taxonomic status of the tested mosquitoes, individual *A. gambiae* specimens were processed following a molecular PCR-RFLP protocol which simultaneously identifies the specific and molecular form status of members of the *A. gambiae* complex [72]. As tested mosquitoes were not half-gravid, we could not score chromosomal inversions by traditional cytogenetic techniques [73]. Instead, we used two DNA-based diagnostic PCR assays for the 2L*a* [25] and 2R*b* [26] inversions. While the diagnostic test for 2L*a* is reliable, the one for 2R*b* provides 81–96% congruence with cytogenetic techniques depending on karyotype, making inferences concerning the effect of this inversion less trustworthy. Molecular assays were carried out either directly on one mosquito leg or on genomic DNA extracted from a leg or the thorax by the CTAB-DNA method (modified from Weeks *et al.* [74]).

### Assessment of Body Size

Body mass (which represents a combination of the body’s linear dimensions and body water stores) significantly affects the survival of mosquitoes in a desiccating environment [50]. Logistic constraints prevented us from measuring the mass of individual mosquitoes; we therefore measured wing length as a proxy for body mass and assumed a strong relationship between the two variables since all mosquitoes were reared in identical conditions [75]. Additionally, body water content and dry body mass are strongly correlated at emergence (r^2^ = 0.86, data from Gray *et al*., [29]), suggesting that wing length measurements provide an adequate correlate of overall body size and associated water stores.

After death, a wing of each specimen was removed, mounted on a microscope slide, and fastened with a cover slide using thin transparent adhesive tape. We measured the distance between the alula and the distal end (excluding the fringe) of the left wing–or the right wing whenever the left one was damaged–under a dissecting microscope. A picture of the wing was taken with a microscope video camera, and the image analysed using measurement tools available in the software Motic Image v.2.0.

### Statistical Analysis

Because of non-normal data (Shapiro-Wilk test: W = 0.9886, *P* = 0.001), wing lengths were transformed prior to analysis according to the Box-Cox algorithm with the parameter lambda estimated from the data at –0.75. This transformation normalised the data (Shapiro-Wilk test: W = 0.9972, *P* = 0.664). To test for differences in body size among karyotypes, wing lengths were subjected to a linear mixed-model ANOVA using karyotype status and sex as fixed effects and replicates as random effects.

Preliminary analyses showed that martingale residuals in survival analysis behaved better when the variable wing length was replaced by a cubic transformation of wing length, which we defined as SIZE  =  (WING LENGTH)^3^. This makes intuitive sense as it is the volume (linear dimension cubed) more so than the linear dimension of the animal that should affect survival in a dry environment. Indeed, the Cox regression model including SIZE instead of WING LENGTH returned a lower AIC (2374.3 instead of 2375.8). Further analyses were therefore carried out with the SIZE variable instead of WING LENGTH.

Non-parametric survival regression analysis was performed to examine the association between chromosomal arrangements and survival. Given the presence of some censored observations, survival distributions were represented by Kaplan–Meier survivorship curves, and modelled using Cox proportional hazards models stratified by REPLICATE, considering time to death (in min) as the response variable and three explanatory variables: SIZE (i.e. wing length cubed), SEX (males *vs*. females) and KARYOTYPE status (standard homokaryotype, heterokaryotype, or inverted homokaryotype).

The Akaike Information Criterion (AIC) provided assessment of the minimal adequate models; the approximate probability of significance of individual model terms was further evaluated by incremental removal of model terms and likelihood-ratio tests following the procedures described in [76], [77]. All analyses were performed in the statistical software « R » v.2.9.0 [78], using the additional libraries « *survival* » [79] and «*nlme»* [80].

## Supporting Information

Figure S1 **Survival of** ***Anopheles gambiae*** **mosquitoes submitted to desiccation stress.** Kaplan-Meier survivorship curves stratified according to (A) wing length; and (B) replication (cohort of emerging mosquitoes). Wing length was subdivided in four classes corresponding to intervals of length defined by quartiles as in Table 1 of main text.
(TIF)

Figure S2 **Relative contribution of 2L** ***a*** **karyotype and body size on** ***Anopheles gambiae*** **survival under desiccation stress.** Contour plot showing isoclines of relative death hazard for mosquitoes of varying wing length (on the abscissa) compared to individuals whose wing length differs from that of the reference length shown on the abscissa by the amount plotted on the ordinate.
(TIF)

Table S1 **Statistical inference (table above) and parameter estimates (table below) of Generalised Linear Models assessing the impact of sex and karyotype status on wing length.**
(PDF)

Table S2 **Cox proportional hazards models of mosquito survival under desiccation stress.**
(PDF)

Text S1 **Details of the statistical analysis pertaining to the Generalised Linear Models assessing the impact of sex and karyotype status on wing length, presented in Table S1.**
(PDF)

Text S2 **Details of the statistical analysis pertaining to the Cox proportional hazards models, presented in Table S2.**
(PDF)

Text S3 **Discussion of the relative contribution of karyotype status and size to** ***Anopheles gambiae*** **survival submitted to desiccation resistance tests, presented in Fig. S2.**
(PDF)
