# Supplementary figures and images for: Adaptation to Aridity in the Malaria Mosquito Anopheles gambiae: Chromosomal Inversion Polymorphism and Body Size Influence Resistance to Desiccation
Source: PLoS One. 2012 Apr 13;7(4):e34841. doi: 10.1371/journal.pone.0034841 (PMC3325948; doi:10.1371/journal.pone.0034841)

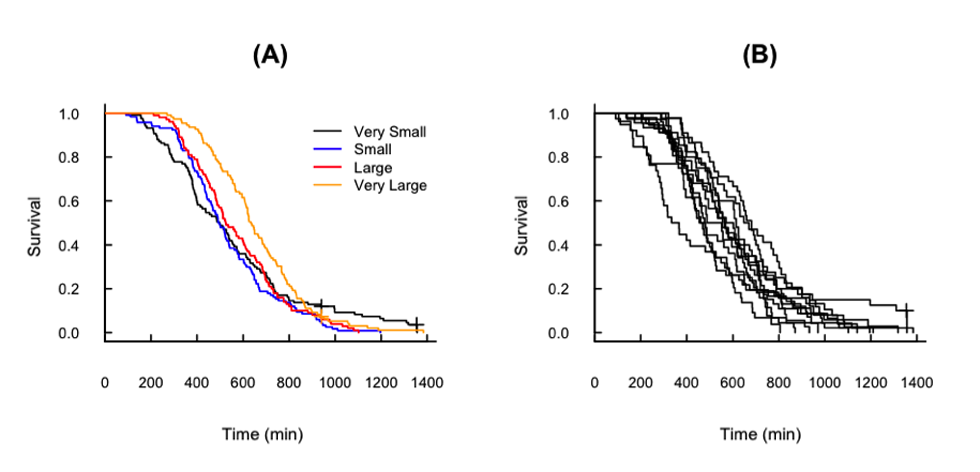

Supplement: Figure S1 — Survival of Anopheles gambiae mosquitoes submitted to desiccation stress. Kaplan-Meier survivorship curves stratified according to (A) wing length; and (B) replication (cohort of emerging mosquitoes). Wing length was subdivided in four classes corresponding to intervals of length defined by quartiles as in Table 1 of main text. (TIF) [file pone.0034841.s001.tif]

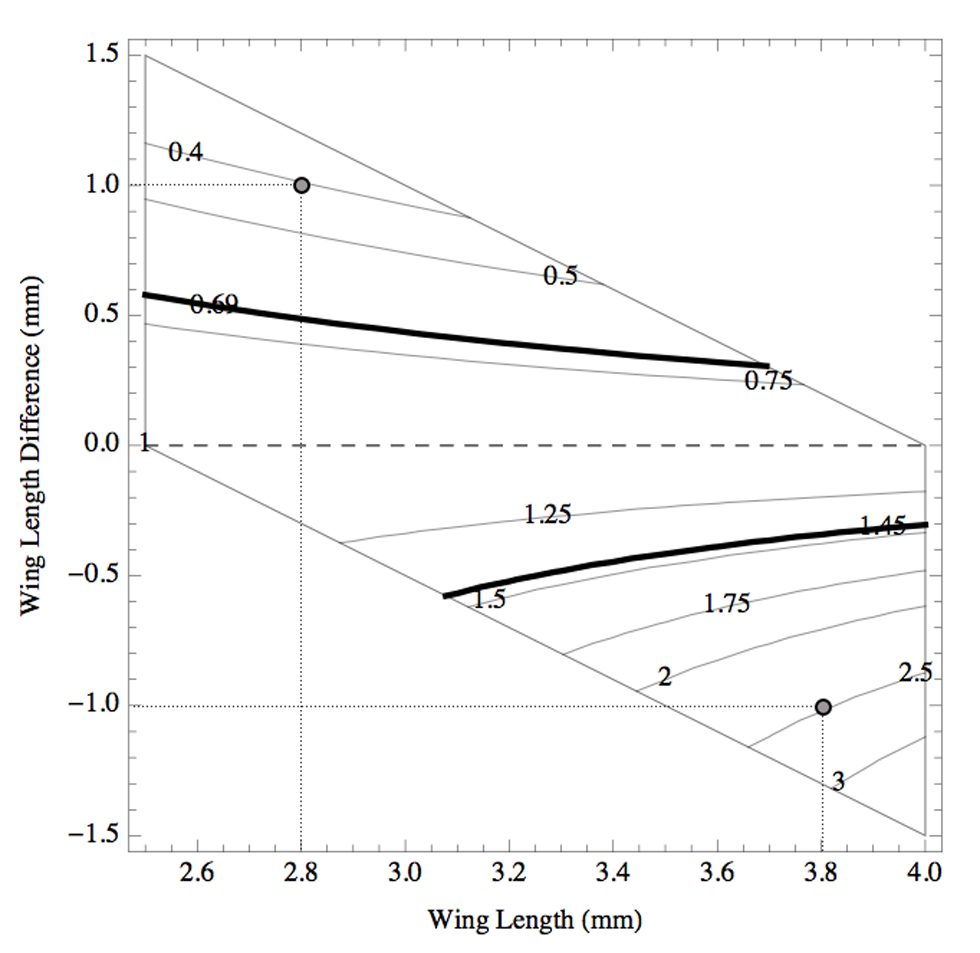

Supplement: Figure S2 — Relative contribution of 2L a karyotype and body size on Anopheles gambiae survival under desiccation stress. Contour plot showing isoclines of relative death hazard for mosquitoes of varying wing length (on the abscissa) compared to individuals whose wing length differs from that of the reference length shown on the abscissa by the amount plotted on the ordinate. (TIF) [file pone.0034841.s002.tif]
