# Supplementary material for: Adaptation to Aridity in the Malaria Mosquito Anopheles gambiae: Chromosomal Inversion Polymorphism and Body Size Influence Resistance to Desiccation
Source: PLoS One. 2012 Apr 13;7(4):e34841. doi: 10.1371/journal.pone.0034841 (PMC3325948; doi:10.1371/journal.pone.0034841)
Supplement: Table S1 — Statistical inference (table above) and parameter estimates (table below) of Generalised Linear Models assessing the impact of sex and karyotype status on wing length. (PDF) [file pone.0034841.s003.pdf]

Variables in the model	AIC	LR test	d.f.	*P*
1. Null Model	-1829.1	7.57	3	0.056
2. KARYOTYPE	-1811.3	21.39	1	<0.001
3. SEX	-1849.0	14.38	2	<0.001
4. KARYOTYPE + SEX	-1830.7	12.07	2	0.002
5. Main Effects + KARYOTYPE*SEX	-1814.6			

Statistical inference evaluators used to identify the minimal adequate model: Akaike Information Criterion (AIC), Likelihood Ratio (LR) tests and associated approximate probability (*P*) values. The null model includes no variables. LR tests quantify the increase in deviance caused by the removal of a variable or an interaction between different variables from a model. The more appropriate way to perform LR tests is to fit first more complex models and then remove variables (or interactions) one-by-one from the more complex model of the same order; if the removal of a variable (or interaction) does not produce a statistically significant increase in deviance, then it is assumed that it is not justified to include that variable (or interaction) in the model. For example, the comparison between Models 2 and 4 indicates that removal of the variable SEX from Model 4 produced a statistically significant increase in deviance (LR test=21.39; d.f=1; *P*<0.001).

Parameter	Estimate	SE	t-value	*P*
(Intercept)	0.7751	0.0064	121.14	<0.0001
SEX:Males	-0.0185	0.0055	-3.35	0.0009
KARYOTYPE:Inverted	0.0043	0.0055	0.77	0.4409
KARYOTYPE:Standard	-0.0084	0.0046	-1.82	0.0691
SEX:Males*KARYOTYPE:Inverted	-0.0079	0.0086	-0.92	0.3583
SEX:Males*KARYOTYPE:Standard	0.0068	0.0065	1.05	0.2956

Parameter estimates, standard errors (SE), and statistical significance of the maximal model having all main effects and their interaction (Model No. 5). Parameter estimates are expressed as a difference with respect to the baseline value represented by 2L*a*-heterozygous females (i.e. the intercept).
