# Supplementary material for: Adaptation to Aridity in the Malaria Mosquito Anopheles gambiae: Chromosomal Inversion Polymorphism and Body Size Influence Resistance to Desiccation
Source: PLoS One. 2012 Apr 13;7(4):e34841. doi: 10.1371/journal.pone.0034841 (PMC3325948; doi:10.1371/journal.pone.0034841)
Supplement: Table S2 — Cox proportional hazards models of mosquito survival under desiccation stress. (PDF) [file pone.0034841.s004.pdf]

Variables in the model	AIC	LR test	d.f.	*P*
1. Null Model	2390.1			
2. KARYOTYPE	2383.0	16.76	2	<0.001
3. SEX	2385.3	21.10	3	<0.001
4. SIZE	2374.1	9.85	3	0.020
5. KARYOTYPE + SEX	2380.1	11.90	1	0.001
6. KARYOTYPE + SIZE	2369.8	1.59	1	0.207
7. SEX + SIZE	2373.6	7.35	2	0.025
8. KARYOTYPE + SEX + SIZE	2370.2	8.02	5	0.155
9. Main Effects + KARYOTYPE*SEX	2371.1	2.90	2	0.235
10. Main Effects + KARYOTYPE*SIZE	2373.9	5.65	2	0.059
11. Main Effects + SEX*SIZE	2373.9	5.65	2	0.059
12. Main Effects + KARYOTYPE*SEX + KARYOTYPE*SIZE	2372.2	1.98	1	0.159
13. Main Effects + KARYOTYPE*SEX + SEX*SIZE	2371.1	2.90	2	0.235
14. Main Effects + KARYOTYPE*SIZE + SEX*SIZE	2373.9	5.65	2	0.059
15. Main Effects + KARYOTYPE*SEX + KARYOTYPE*SIZE + SEX*SIZE	2372.2	1.97	2	0.373
16. FULL MODEL (Main Effects + All 2nd and 3rd-order Interactions)	2374.3			

Statistical inference evaluators used to identify the minimal adequate model: Akaike Information Criterion (AIC), Likelihood Ratio (LR) tests and associated approximate probability (*P*) values. The null model includes no variables. LR tests quantify the increase in deviance caused by the removal of a variable or an interaction between different variables from a model. The more appropriate way to perform LR tests is to fit first more complex models and then remove variables (or interactions) one-by-one from the more complex model of the same order; if the removal of a variable (or interaction) does not produce a statistically significant increase in deviance, then it is assumed that it is not justified to include that variable (or interaction) in the model. For example, the comparison between Models 6 and 8 indicates that removal of the variable SEX from Model 8 did not produce a statistically significant increase in deviance (LR test=1.59; d.f=1; P=0.207).
