# Supplementary material for: Adaptation to Aridity in the Malaria Mosquito Anopheles gambiae: Chromosomal Inversion Polymorphism and Body Size Influence Resistance to Desiccation
Source: PLoS One. 2012 Apr 13;7(4):e34841. doi: 10.1371/journal.pone.0034841 (PMC3325948; doi:10.1371/journal.pone.0034841)
Supplement: Text S1 — Details of the statistical analysis pertaining to the Generalised Linear Models assessing the impact of sex and karyotype status on wing length, presented in Table S1. (PDF) [file pone.0034841.s005.pdf]

Text S1. Details of the statistical analysis pertaining to the Generalised Linear Models assessing the impact of sex and karyotype status on wing length, presented in Table S1 of Supporting Information.

Several linear mixed models having SEX and KARYOTYPE as fixed effects and REPLICATION as a random effect were evaluated by a set of statistical evaluators (Table S1). The minimal adequate model, as inferred by the lowest Akaike Information Criterion (AIC), was the one having only the factor SEX included as explanatory variable (Model 3 in Table S1). However, the removal of the SEX*KARYOTYPE interaction from the full model (Model 4 in Table S1) provoked a significant (P=0.002) increase in deviance, as shown from the likelihood ratio (LR) test comparing Model 4 *vs*. Model 5. On the other hand, inspection of the parameter estimates of the maximal model (Model 5) showed that the individual parameter estimates of the interaction between SEX and KARYOTYPE were not statistically significant (Table S1). The removal of the main effect KARYOTYPE caused a significant increase in deviance (LR test, Model 3 *vs*. Model 4, P=0.001; Table S1). We conclude, therefore, that evidence for a significant association between female carriers of the 2L*a* inversion and wing length (i.e. a significant interaction between SEX and KARYOTYPE) is not strong enough in our data set.
