# Supplementary material for: Adaptation to Aridity in the Malaria Mosquito Anopheles gambiae: Chromosomal Inversion Polymorphism and Body Size Influence Resistance to Desiccation
Source: PLoS One. 2012 Apr 13;7(4):e34841. doi: 10.1371/journal.pone.0034841 (PMC3325948; doi:10.1371/journal.pone.0034841)
Supplement: Text S2 — Details of the statistical analysis pertaining to the Cox proportional hazards models, presented in Table S2. (PDF) [file pone.0034841.s006.pdf]

Text S2. Details of the statistical analysis pertaining to the Cox proportional hazards models, presented in Table S2 of Supporting Information.

Table S2 presents the values of the statistical evaluators used to gauge the significance of covariables upon survival. The model having the lowest AIC was that with KARYOTYPE and SIZE as main effects (Model 6). Some of the second-order interactions, however, were marginally non-significant ($P \approx 0.06$) as judged by Likelihood Ratio tests after removal from the model having all interactions of the same order (e.g. SEX*KARYOTYPE, KARYOTYPE*SIZE, and SEX*SIZE interaction terms from Model 15). Given the relationship existing between sex and karyotype with size (Fig. 2), and the strong effect of size upon desiccation resistance (Fig. S1A), it is possible that these LR tests gave marginally significant values due to the correlation existing between these variables. Thus, we resorted to the AIC criterion to identify Model 6 as the minimal adequate model (Table S2).
