# Supplementary material for: Adaptation to Aridity in the Malaria Mosquito Anopheles gambiae: Chromosomal Inversion Polymorphism and Body Size Influence Resistance to Desiccation
Source: PLoS One. 2012 Apr 13;7(4):e34841. doi: 10.1371/journal.pone.0034841 (PMC3325948; doi:10.1371/journal.pone.0034841)
Supplement: Text S3 — Discussion of the relative contribution of karyotype status and size to Anopheles gambiae survival submitted to desiccation resistance tests, presented in Fig. S2. (PDF) [file pone.0034841.s007.pdf]

Text S3. Discussion of the relative contribution of karyotype status and size to *Anopheles gambiae* survival submitted to desiccation resistance tests, presented in Fig. S2 of Supporting Information.

To compare the relative contribution of karyotype status and mosquito size measured on the linear scale of wing length, we plotted the estimated death hazard for a range of reference wing length values in relation to increasing or decreasing values of length relative to the reference length (Fig. S2). The region in the plot lying between the 0.69 and 1.45 contour lines, representing the hazard ratio due to karyotype status, outlines the range of wing lengths and wing length differentials where the effect of karyotype status upon survival was greater than the effect of mosquito size. It can be seen from the plot that the effect of karyotype was greater for smaller mosquitoes and lower wing length differentials. At higher values of wing length or wing length differentials, the effect of mosquito size became stronger than that of 2L*a* karyotype status.

For example, the point in Fig. S2 lying next to the isocline on the top left corner of the plot, denotes that mosquitoes having a wing length of 3.8 mm (2.8+1.0 mm) had a death hazard ratio of *c.* 0.4 compared to mosquitoes whose wing length is 1.0 mm shorter (i.e. 2.8 mm). In a complementary way, mosquitoes whose wing length is 2.8 mm (3.8–1.0 mm) had a hazard ratio of *c.* 2.5 (i.e. 1/0.4) compared to those whose wing length is 1.0 mm longer, that is, 3.8 mm (shown by the point lying on the bottom right corner of the plot). The boundary inside the plot outlines the range of wing lengths and wing length differentials recorded in our study population. The dashed line identifies a hazard ratio of one, i.e. equal survival, corresponding to pairs of individuals whose wing length difference is equal to zero. The thicker isoclines identify the death hazard ratio of 0.69, and its inverse 1.45 (1/0.69), which correspond to the adjusted hazard ratio due to 2L*a* karyotype status (cf. ψ values in Table 2 of main text). Accordingly, points lying inside the region defined by the thicker isoclines and the plot boundaries correspond to pairs of individuals for which size (i.e. wing length) contributed less than karyotype status to desiccation resistance.
